# Social, Environmental and Psychological Factors Associated with Objective Physical Activity Levels in the Over 65s

**DOI:** 10.1371/journal.pone.0031878

**Published:** 2012-02-16

**Authors:** Marion E. T. McMurdo, Ishbel Argo, Iain K. Crombie, Zhiqiang Feng, Falko F. Sniehotta, Thenmalar Vadiveloo, Miles D. Witham, Peter T. Donnan

**Affiliations:** 1 Ageing and Health, University of Dundee, Dundee, Tayside, Scotland; 2 Division of Population Health Sciences, University of Dundee, Dundee, Tayside, Scotland; 3 School of Geography and Geosciences, University of St Andrews, St Andrews, Fife, Scotland; 4 Centre for Translational Research in Public Health, University of Newcastle, Newcastle, England; Wayne State University, United States of America

## Abstract

**Objective:**

To assess physical activity levels objectively using accelerometers in community dwelling over 65 s and to examine associations with health, social, environmental and psychological factors.

**Design:**

Cross sectional survey.

**Setting:**

17 general practices in Scotland, United Kingdom.

**Participants:**

Random sampling of over 65 s registered with the practices in four strata young-old (65–80 years), old-old (over 80 years), more affluent and less affluent groups.

**Main Outcome Measures:**

Accelerometry counts of activity per day. Associations between activity and Theory of Planned Behaviour variables, the physical environment, health, wellbeing and demographic variables were examined with multiple regression analysis and multilevel modelling.

**Results:**

547 older people (mean (SD) age 79(8) years, 54% female) were analysed representing 94% of those surveyed. Accelerometry counts were highest in the affluent younger group, followed by the deprived younger group, with lowest levels in the deprived over 80 s group. Multiple regression analysis showed that lower age, higher perceived behavioural control, the physical function subscale of SF-36, and having someone nearby to turn to were all independently associated with higher physical activity levels (R^2^ = 0.32). In addition, hours of sunshine were independently significantly associated with greater physical activity in a multilevel model.

**Conclusions:**

Other than age and hours of sunlight, the variables identified are modifiable, and provide a strong basis for the future development of novel multidimensional interventions aimed at increasing activity participation in later life.

## Introduction

Regular physical activity is an important determinant of health and function in later life [1]. Despite decades of research our understanding of the factors that are important in promoting regular physical activity is still in its infancy [2], [3]. In consequence current approaches to promoting activity have been disappointing. Inactivity is particularly common amongst older people, and only 7% of men and 4% of women over the age of 75 years in the UK reach current physical activity recommendations [4]. Widely available exercise referral schemes together with walking, cycling and pedometer schemes have insufficient evidence to support their use and the UK National Institute for Health and Clinical Excellence (NICE) advises such approaches should be used only in the context of properly designed research studies.

Progress with effective physical activity promotion has suffered from a focus on individual-level characteristics and behaviours [5], [6]. However the physical activity behaviours of older people are likely to vary according to a range of individual, social, and environmental factors. Such factors have been incompletely characterised, and the interactions between them have been largely ignored. Psychological research has shown that perceptions of positive and negative consequences (attitudes), social approval (subjective norm), and capability (perceived behavioural control), all influence decision making and behaviour [7]. These social cognitions can be modified by appropriate behavioural techniques.

Good social networks are essential for successful ageing [8]. Older people are at high risk of losing critical social ties as they age and both social support and social capital are independently linked to poor self-rated health. The physical and residential environments in which older people live (e.g. proximity of shops, post offices) are under-explored influences on opportunities for physical and social activity [9], [10]. Moreover psychological, social and environmental factors might interact; for example the effects of the environment on physical activity might be buffered through social support [11].

We therefore designed a study to determine which individual, social and environmental factors explain person to person variation in daily physical activity in older people, with a view to designing a future intervention to increase activity participation.

## Methods

### Study population and recruitment

We performed a prospective, cross-sectional survey on a stratified sample of community-dwelling older people, resident in Tayside, Scotland. Participants were eligible for inclusion if they were aged 65 or over and were ambulant with or without walking aids. Potential participants were excluded if they were resident in institutional care (hospital, nursing or care home), were unable to give written informed consent, were unwilling to participate or were participating in another research study.

We recruited participants from 17 primary care practices in Tayside. Practices were purposively selected from the Scottish Primary Care Research Network to give a spread of rural vs urban areas, and affluent vs deprived areas. For each practice, the Scottish Primary Care Research Network drew random samples of eligible subjects stratified by age (65–80 years; age>80 years) and Scottish Index of Multiple Deprivation (http://www.isdscotland.org) based on postcode (SIMD deciles 1–4 ‘deprived’, SIMD 5–10 deciles ‘affluent’), in order to ensure adequate representation of deprived and very old participants. Each practice reviewed the list generated and excluded from it anyone that it would be unsuitable to contact. Potential participants were invited to opt in to the study. Those responding positively were telephoned, their eligibility was confirmed, and an appointment made for a home visit. Potential participants not responding, or declining the invitation, were replaced by another randomly selected individual from the same stratum.

We obtained written informed consent from participants and the study was approved by the Tayside Committee on Medical Research Ethics (09/S1401/57). Participants were not compensated for participation. The study conformed to the principles of the Declaration of Helsinki.

### Assessment schedule

We conducted two home visits with each participant, spaced 7 days apart. Details of the questionnaires administered at each visit are given in Table 1. At the baseline visit, we provided participants with an RT3 accelerometer (Stayhealthy inc, Monrovia, California, USA). The RT3 is a piezoelectric, triaxial accelerometer which has previously been shown to discriminate walking from sedentary activity in older people [12], and which is responsive to interventions designed to increase physical activity [13]. Participants wore the accelerometer on the waistband anteriorly over the same hip during waking hours for a single 7-day period [14]. Summed activity counts were recorded each minute for 7 days. 24 hour periods commenced at midnight; the partial data from the first and last day was therefore discarded.

**Table 1:** Questionnaires administered to participants.

*Baseline visit*	*Domain measured*
Hospital Anxiety and Depression Score (HADS) [28]	Depression, Anxiety
SF-36 [29], [30]	Health-related quality of life
Functional Limitations Profile [31]	Self-reported physical and psychosocial function
*Extended Theory of Planned Behaviour questionnaire [32]	Attitudes, beliefs, intentions around physical activity
London Health and Fitness questionnaire [33]	Current and previous self-reported activities, attitudes to exercise
*1 week visit*	
R-UCLA loneliness scale [34]	3 item self-reported loneliness
Social Capital module [35]	Self-reported perceptions of connectedness to relatives/friends/sources of help
Older Peoples Active Living (OPAL) questionnaire [36]	Self-reported perceptions of surroundings (facilities, safety, social, transport) and general health questions

*Examples of questions relating to perceived behavioural control included:

“To what extent do you see yourself as being able to do 30 minutes of moderate-intensity physical activity on 5 or more days in the coming week?”.

“How confident are you that you will be able to do 30 minutes of moderate-intensity physical activity on 5 or more days in the coming week?”.

We provided seven-day activity diaries in which participants recorded minutes of outdoor activity per day (morning, afternoon, evening) and location of activity, to identify what types of outdoor environments people spent their time in.

Global Positioning System (GPS) technology (Garmin Etrex-H, Garmin, Olathe, Kansas, USA) was used to obtain latitude and longitude coordinates for each participants' home. Gradients around each home were calculated using ArcGIS 9.3 (Esri Inc, Redlands, California, USA) based on 50 m digital elevation model (DEM) from Ordnance Survey [15]. Daily regional hours of sunlight, rainfall and maximum temperature data were obtained from the Meteorological Office, utilising data collected at the Mylnefield weather station. All participants lived within 30 miles of the weather station.

Post office, grocery store, supermarket locations in the form of postcode were obtained from the yellow pages (http://www.yell.com). These postcodes were then converted into geographical coordinates with a precision of one metre using the postcode directory [16]. The OS ITN (Ordnance Survey Integrated Transport Network) dataset, acquired under an academic license from EDINA/Digimap, was used in calculation of distances to postcode offices. The OS ITN is a vector database consisting of motorway, A-road, B-Road and minor road features. Road distances from respondents' residence to the nearest post office, grocery store or supermarket were calculated using ArcGIS Network Analyst. These distances were represented in kilometres. Green spaces were defined as open, undeveloped land with national vegetation and included, for example, parks, forests, playing fields and river corridors. Green spaces near residence were measured in percentage of green space in the census ward (approximately 5000 people) where a respondent lived. The data was obtained from http://cresh.org.uk/ [17].

### Data handling

Data were collated, anonymised and processed by the Health Informatics Centre (HIC, http://www.dundee.ac.uk/hic) according to their standard operating procedures. A 5% random sample was entered twice to assess errors and the mean error rate was 0.8%.

### Sample Size and Statistical Methods

We planned a sample size of 600 (150 per stratum) to permit the influence of an individual attribute to be detected with 80% power if the correlation with a particular outcome was as low as 0.11. For multiple regression this sample size would allow consideration of 30 variables with a multiple correlation coefficient of 0.2 or R-squared of 4%. It would allow detection of a change in R-squared as low as 2.5% in a multiple regression model with the addition of 15 variables to a model with 15 variables already entered with 80% power.

Data for each outcome were summarised by the descriptive statistics of mean and standard deviation if normally distributed and as percentages for categorical variables. Correlations between variables were estimated to assess co-linearity between questionnaire items and across questionnaires. A two-sided p value of 0.05 was taken to denote significance unless otherwise stated. A series of regression models were constructed with physical activity (mean activity counts per 24 hour period for each participant) as the outcome stratified by age and SIMD. Initially, univariate regression analyses were carried out and only factors meeting the Hosmer-Lemeshow criterion of univariate p<0.3 proceeded to multiple regression. Factors were grouped as psychological, social, environmental or demographic and these groups of factors were analysed separately. From these models a final model combining significant factors from each group was constructed. To test the effect of variables changing on a daily basis (e.g. weather data), mixed modelling was employed, using each 24 hour activity count as the dependent variable. No adjustment was made for geographic clustering in the mixed model. For this final model weather variables of sunlight hours, rainfall and temperature were entered as level 1 variables, with other social, environmental, psychological and demographic variables entered as level 2 variables. Analyses were carried out using SPSS v18 (SPSS inc, Chicago, USA) and SAS v9.2 (SAS, Cary, North Carolina, USA).

## Results

Participants were recruited between October 2009 and January 2011 from 17 General Practices throughout Tayside across a spread of rural and urban areas.

### Response rates

3343 letters of invitation were sent to potential participants. The deprivation category was unknown for 7/3343 so 3336 was used as the denominator. Overall 63% replied to their invitation (either positively or negatively) and 19% responded positively indicating a willingness to participate.

A total of 584 people, age range 65 to 100 years, 46% (268/584) male participated, i.e. 97.3% (584/600) of the target sample size. Table S1 shows their descriptive characteristics for those with complete data by deprivation category and age group. The intention had been to recruit 150 per group, but as expected the over 80 s proved more difficult to recruit, particularly those from more deprived areas. This was despite oversampling those in the more deprived groups.

Participants varied little by age in the descriptive characteristics, but there were major differences by deprivation: the affluent were much more likely to live in small towns and rural areas and had more access to green space.

### Drop outs and missing data

580/584 (99.3%) took part in both visits. This was because 2 participants fell shortly after the baseline visit, one participant was diagnosed with metastatic cancer, and one participant cancelled his 7 day visit and was not subsequently contactable.

Accelerometry data were available for 547/584 (93.6%) of participants. Accelerometry data were missing for the remaining participants for various largely technical reasons for example, difficulties downloading the data or the battery becoming dislodged.

### Activity measurement

The mean (SD) number of minutes of activity per day recorded by accelerometry were highest at 214 (89) for the affluent young-old; followed by 192 (84) for the deprived young-old, deprived; then 156 (87) for the affluent old-old; and lowest 128 (68) for the deprived old-old. For the total population of 547 participants the mean (SD) was 177 (89) minutes per day. A similar pattern was found for counts of activity per day. Because the correlation between accelerometry counts and minutes was very high (correlation coefficient 0.967, p<0.001), analysis of count data only are subsequently presented.

The daily diary entries recorded the number of minutes perceived by the participant to be time spent in outdoor physical activity on a daily basis. The sum of the minutes in the diary was only modestly correlated with both the accelerometer counts (Spearman's rho = 0.256, p<0.01) and with accelerometer minutes (Spearman's rho = 0.238, p<0.01).

### Univariate regression analysis

Table S2 presents the variables which showed significant associations with physical activity, the percentage of variance explained (R^2^) as well as the p-values. Modelling results were similar for both counts of activity and minutes of activity from accelerometry so subsequent regression models were implemented on physical activity counts. Significant predictors were found in each of the domains studied: age, physical environment, physical functioning, functional limitations, mental well being and social and psychological factors. As expected, younger age and high physical functioning were strongly associated with activity levels.

Psychological predictors i.e. behavioural beliefs (perceived incentives and disincentives), normative beliefs (perceived approval of important others), perceived behavioural control (self efficacy) and intention showed strong positive associations with physical activity levels.

### Multiple regression analysis

Although many perceptions of social influences, such as neighbourliness and availability of help in a crisis, were significant, the size of these relationships was modest.

As many of the significant univariate factors were correlated (data not shown), stepwise multiple linear regression was used to identify those which exerted independent statistically significant effects. Initially, all social and environmental factors that failed to pass the Hosmer-Lemeshow criterion of p<0.3 were eliminated. In the final model, increasing age remained one of the strongest negative associations with physical activity (Table 2). Physical functioning as measured in the SF36 was also strongly associated with physical activity independently of age. After adjustment for these two factors only two further variables independently accounted for additional variability in physical activity levels; namely, perceived behavioural control and the ‘number of people that participants could turn to nearby’. Participants with high perceived behavioural control and relevant others nearby were more physically active.

**Table 2:** Factors showing independent statistically significant associations with the outcome of mean physical activity counts* (n = 547).

Variable	Beta	SE	t	Significance
Intercept	252114	36928	6.83	<0.001
Age (+1 yr)	−2835	430	−6.59	<0.001
PBC (High vs Low)	4523	1619	2.79	0.005
Physical functioning (SF36) (+1 unit)	1122	160	6.99	<0.001
No. people can turn to nearby (log)	19506	9388	2.08	0.038

*In stepwise linear regression. Stratified by age group and SIMD. Adjusted R^2^ = 0.32.

PBC – perceived behavioural control.

### Mixed model analysis of daily activity counts

Finally, in order to assess factors that varied on a daily basis, a mixed model was implemented (Table 3). A significant interaction between perceived behavioural control and age was found, where the beneficial effect of perceived behavioural control waned with increasing age. Mean counts for ‘low’ perceived behavioural control were 114434 (95% CI 105906, 122961) and ‘high’ perceived behavioural control (self efficacy) were 165935 (95% CI 157137, 174733). Finally, as daily activity measures were assessed in the mixed model, weather variables were also considered and increased hours of sunlight were significantly associated with increasing activity, independently of the other significant factors.

**Table 3:** Results of linear mixed model on daily physical activity counts* (n = 3207).

Variable	Beta	SE	t	Significance
Intercept	206168	70764	2.91	0.004
Age (+1 yr)	−2063.8	818.3	−2.52	0.012
Day of measurement (+1 day)	−2247.0	553.1	−4.06	<.0001
Average Hours of sunlight (+1 hr)	317.6	132.7	2.39	0.017
PBC (High vs low)	139241	61734	2.26	0.024
Age×PBC interaction	−1596.9	776.2	−2.06	0.040
Physical function (SF36) (+1 unit)	1239.7	155.3	7.99	<.0001
No. people could turn to nearby (log)	19733	9422	2.09	0.036

*Stratified by age group and SIMD.

PBC – perceived behavioural control.

Table 4 shows physical activity counts by ‘high’ and ‘low’ perceived behavioural control.

**Table 4:** Mean (SD) Physical Activity Count by Perceived Behavioural Control (High, Low) and by quartile of age.

	Perceived behavioural control	
Age Quartile	Low	High
1 (Youngest)	143,365 (68,764)	206,217 (82,831)
2	116,145 (49,574)	169,236 (77,467)
3	130,751 (75,451)	142,255 (71,670)
4 (Oldest)	79,806 (51,439)	121,672 (71,014)

Tests of every possible interaction of age with the significant variables were explored in the final model. All were non-significant (data not shown) apart from the significant age by perceived behavioural control interaction.

## Discussion

We have conducted what is to our knowledge, the largest European study to date to have objectively measured physical activity levels in an older population [18], [19]. It is the first to have comprehensively studied the interactions between individual, social, behavioural and environmental influences on physical activity in older people. We found that physical activity was independently associated with younger age, greater social support, more average hours of sunshine, higher perceived behavioural control and physical function from SF-36. In addition, an interaction between perceived behaviour control and age indicated that perceived behavioural control might be less beneficial in older age. Although the influence of perceived behavioural control declined with increasing age, those with high perceived behavioural control had higher activity counts than those with low perceived behavioural control.

A strength of this study is that substantial proportion of our sample (42%) were over the age of 80 years. The over 80 s are an important, growing but neglected group in the research literature, and scant data exist on how to promote physical activity for this group [20]. Our study also benefits from the use of a wide range of validated instruments with which to measure the factors predicting physical activity.

A further strength is the use of an objective measure, accelerometry, to assess physical activity. We found that there was only poor correlation between objective and self-reported activity. The limitations of self report include social desirability bias, poor sensitivity and dependence on recall [21]. Our results are consistent with the US National Health and Nutrition Examination Study (NHANES) study which found that self-reported physical activity data significantly overestimated physical activity count, duration and adherence [22]. Whilst previous smaller studies have used accelerometry to record activity levels in older people their findings are limited by recruitment from a single, middle income practice [23], and by a focus exclusively on urban-dwelling older people [24]. Our study is both large and covers urban and rural dwelling people.

A limitation of this study is the extent to which our findings generalise to the whole of the UK and to other developed countries. It is possible that our estimates of activity levels may differ from those in other parts of the UK. However such factors are unlikely to affect the relationships between psychological, social and environmental factors and physical activity.

We confirmed associations from other studies between lower physical activity levels in later life and increasing age, disability/physical function and self efficacy [24]. However we also examined seasonal, environmental and psychological factors and modeled their relationships with activity levels. Our results are unique in also identifying that having someone nearby to whom one can turn to and average hours of sunshine both independently add to the prediction of physical activity. Having someone nearby to turn might be a motivator to leave their house and thereby be more socially and physically active. Low physical activity may be explained by a lack of reason to walk by virtue of simply not having people nearby that one would wish to visit.

Day length and weather conditions are known to strongly influence physical activity participation in frail older people [25] but in our survey the effect of hours of sunshine was independently associated with physical activity, while temperature was not.

Our analysis of psychological factors found that all variables showed substantial associations with objectively recorded physical activity. This is in keeping with the literature, but in contrast to our findings in a recent trial of pedometer use in older women [13].

Our study has identified important opportunities for interventions to increase levels of physical activity, particularly perceived behavioural control and the ‘number of people that participants could turn to nearby’. A range of interventions aimed at promoting social contacts exist including befriending and ‘buddy’ schemes [26]. Similarly a recent meta-analysis has confirmed [27] that several intervention techniques (vicarious experience and feedback techniques) modify perceived behavioural control. The challenge for future research is to determine whether these techniques can improve physical activity in older people.

### Conclusions

Age, hours of sunshine, perceived behavioural control, physical function from SF-36, and social support were all independently associated with physical activity. As part of the effect of age is mediated through behavioural factors, it may be possible to modify the effects of age. This survey has identified three modifiable complementary predictors of physical activity which should be targeted in a new multidimensional approach to increasing activity participation in old age (Table 5).

**Table 5:** What is already known and what this paper adds.

Physical activity (PA) is beneficial to older people's health
Research to date has failed to find effective ways to boost PA participation with a predominant focus on single component interventions
Most previous PA surveys have used non-representative samples, and failed to include key target populations i.e. the over 80 s and the socially deprived
This survey comprehensively examined the physical, social, health and environmental determinants of PA measured by accelerometry in a large sample of older people in both affluent and deprived areas
Higher perceived behavioural control, the physical function subscale of SF-36, younger age and having some to turn to were all independently associated with higher physical activity levels. In addition, hours of sunshine were significantly associated with greater physical activity
This study has identified several modifiable factors which can form the basis of future multicomponent PA interventions

## Supporting Information

Table S1 **Descriptive variables by strata.**
(DOCX)

Table S2 **Univariate regressions on physical activity counts with initial p<0.3 to be considered for multiple regression models.**
(DOCX)

## References

[1] Talbot LA, Morrell CH, Metter EJ, Fleg JL (2002). Comparison of cardiorespiratory fitness versus leisure time physical activity as predictors of coronary events in men aged < or = 65 years and >65 years.. Am J Cardiol.

[2] American College of Sports Medicine Position Stand (1998). Exercise and physical activity for older adults.. Med Sci Sports Exerc.

[3] Baker PRA, Francis DP, Soares J, Weightman AL, Foster C (2011). Community wide interventions for increasing physical activity.. Cochrane Database of Systematic Reviews.

[4] Department of Health. Health Survey for England (2000).

[5] Butler L, Dwyer D (2004). Pedometers may not provide a positive effect on walking.. Health Promot J Australia.

[6] McMurdo ME, Johnstone R (1995). A randomized controlled trial of a home exercise programme for elderly people with poor mobility.. Age Ageing.

[7] Hagger MCNBJ (2002). A meta-analytic review of the theoroes of reasoned action and planned behaviour in physical activity: Predictive validity and the contrbution of additional variables.. Journal of Sport and Exercise Psychology.

[8] Litwin H (2001). Social network type and morale in old age.. Gerontologist.

[9] Abbasi AA, Rudman D, Wilson CR, Drinka PJ, Basu SN (1995). Observations on nursing home residents with a history of hip fracture.. Am J Med Sci.

[10] Cunningham GO, Michael YL (2004). Concepts guiding the study of the impact of the built environment on physical activity for older adults: A review of the literature.. American Journal of Health Promotion.

[11] Van Stralen MM (2010). Determinants of physical actvity among the older adults: a literature review.. Health Psychology Review.

[12] Sumukadas D, Laidlaw S, Witham MD (2008). Using the RT3 accelerometer to measure everyday activity in functionally impaired older people.. Aging Clinical and Experimental Research.

[13] McMurdo ME, Sugden J, Argo I, Boyle P, Johnston DW (2010). Do pedometers increase physical activity in sedentary older women? A randomized controlled trial.. J Am Geriatr Soc.

[14] Kochersberger G, McConnell E, Kuchibhatla MN, Pieper C (1996). The reliability, validity, and stability of a measure of physical activity in the elderly.. Arch Phys Med Rehabil.

[15] Environmental Systems Research Institute. ArcGIS. (9.3). 20-8-0008

[16] ONS. ONS Postcode Directory User Guide ONS (2011).

[17] Richardson EA, Mitchell R (2010). Gender differences in relationships between urban green space and health in the United Kingdom.. Social Science & Medicine.

[18] Troiano RP, Berrigan D, Dodd KW, Masse LC, Tilert T (2008). Physical activity in the United States measured by accelerometer.. Med Sci Sports Exerc.

[19] Tudor-Locke C, Johnson WD, Katzmarzyk PT (2009). Accelerometer-determined steps per day in US adults.. Med Sci Sports Exerc.

[20] Baert V, Gorus E, Mets T, Geerts C, Bautmans I (2011). Motivators and barriers for physical activity in the oldest old: A systematic review.. Ageing Res Rev.

[21] Morie M, Reid KF, Miciek R, Lajevardi N, Choong K (2010). Habitual physical activity levels are associated with performance in measures of physical function and mobility in older men.. J Am Geriatr Soc.

[22] Troiano RP, Berrigan D, Dodd KW, Masse LC, Tilert T (2008). Physical activity in the United States measured by accelerometer.. Med Sci Sports Exerc.

[23] Harris TJ, Owen CG, Victor CR, Adams R, Ekelund U (2009). A comparison of questionnaire, accelerometer, and pedometer: measures in older people.. Med Sci Sports Exerc.

[24] Davis MG, Fox KR, Hillsdon M, Sharp DJ, Coulson JC (2011). Objectively measured physical activity in a diverse sample of older urban UK adults.. Med Sci Sports Exerc.

[25] Sumukadas D, Witham M, Struthers A, McMurdo M (2009). Day length and weather conditions profoundly affect physical activity levels in older functionally impaired people.. J Epidemiol Community Health.

[26] Cattan M, Kime N, Bagnall AM (2011). The use of telephone befriending in low level support for socially isolated older people–an evaluation.. Health Soc Care Community.

[27] Ashford S, Edmunds J, French DP (2010). What is the best way to change self-efficacy to promote lifestyle and recreational physical activity? A systematic review with meta-analysis.. Br J Health Psychol.

[28] Zigmond AS, Snaith RP (1983). The hospital anxiety and depression scale.. Acta Psychiatr Scand.

[29] Ware JE, Gandek B (1998). Overview of the SF-36 Health Survey and the International Quality of Life Assessment (IQOLA) Project.. Journal of Clinical Epidemiology.

[30] Lyons RA, Perry HM, Littlepage BNC (1994). Evidence for the Validity of the Short-Form-36 Questionnaire (Sf-36) in An Elderly Population.. Age and Ageing.

[31] Pollard B, Johnston M (2001). Problems with the Sickness Impact Profile: a theoretically based analysis and a proposal for a new method of implementation and scoring.. Social Science & Medicine.

[32] Hagger MS, Chatzisarantis NLD, Biddle SJH (2002). The influence of autonomous and controlling motives on physical activity intentions within the Theory of Planned Behaviour.. British Journal of Health Psychology.

[33] Rowland L, Dickinson EJ, Newman P, Ford D, Ebrahim S (1994). Look-After-Your-Heart Program - Impact on Health-Status, Exercise Knowledge, Attitudes, and Behavior of Retired Women in England.. Journal of Epidemiology and Community Health.

[34] Hughes ME, Waite LJ, Hawkley LC, Cacioppo JT (2004). A short scale for measuring loneliness in large surveys - Results from two population-based studies.. Research on Aging.

[35] Harper R, Kelly M (2003). Measuring social capital in the United Kingdom.. Office of National Statstics.

[36] Fox KR, Hillsdon M, Sharp D, Cooper AR, Coulson JC (2011). Neighbourhood deprivation and physical activity in UK older adults.. Health Place.

