# Supplementary material for: Social, Environmental and Psychological Factors Associated with Objective Physical Activity Levels in the Over 65s
Source: PLoS One. 2012 Feb 16;7(2):e31878. doi: 10.1371/journal.pone.0031878 (PMC3281090; doi:10.1371/journal.pone.0031878)
Supplement: Table S1 — Descriptive variables by strata. (DOCX) [file pone.0031878.s001.docx]

**Table S1. Descriptive variables by strata**

|  | | **‘Deprived’**  **65 -80 years** | **‘Deprived’**  **>80 years** | **‘Affluent’**  **65-80 years** | **‘Affluent’**  **>80 years** | **Total** |
| --- | --- | --- | --- | --- | --- | --- |
|  | |  |  |  |  |  |
| N (%) | | 165 (30) | 99 (18) | 149 (27) | 134 (25) | **547** |
|  | |  |  |  |  |  |
| Mean Age (SD) | | 73(5) | 86 (5) | 73 (5) | 85 (4) | **79 (8)** |
| Number of prescribed medicines (%) | | 3.1 (1.9) | 3.7 (1.8) | 3.0 (1.8) | 3.7 (1.6) | **3.3 (1.8)** |
| % with hospital admission in past 12 months | | 19 | 14 | 15 | 22 | **18** |
| Gender (%) | Male | 45 | 43 | 46 | 49 | **46** |
|  | Female | 55 | 57 | 54 | 51 | **54** |
| Environmental  factors | Resident in primary cities with a population ≥125,000 (%) | 31 | 53 | 6 | 7 | **22** |
|  | Resident in urban settlements and small towns (%) | 65 | 45 | 30 | 40 | **46** |
|  | Resident in rural area (%) | 4 | 2 | 64 | 53 | **32** |
|  | Mean (SD) road distance from house to post office (km) | 0.9  (0.6) | 1.0  (0.6) | 1.9  (2.0) | 1.9  (2.3) | **1.4**  **(1.7)** |
|  | Proportion of green space in the residential ward (%) | 46 | 48 | 79 | 78 | **63** |
| Time of year recruited  (%) | Jan - Mar | 37 | 22 | 32 | 16 | **28** |
|  | Apr – Jun | 26 | 30 | 21 | 27 | **26** |
|  | Jul – Sep | 6 | 10 | 16 | 47 | **20** |
|  | Oct – Dec | 31 | 38 | 32 | 10 | **27** |

‘Deprived’ SIMD 1-4

‘Affluent’ SIMD 5-10
