# Supplementary material for: Social, Environmental and Psychological Factors Associated with Objective Physical Activity Levels in the Over 65s
Source: PLoS One. 2012 Feb 16;7(2):e31878. doi: 10.1371/journal.pone.0031878 (PMC3281090; doi:10.1371/journal.pone.0031878)
Supplement: Table S2 — Univariate regressions on physical activity counts with initial p<0.3 to be considered for multiple regression models. (DOCX) [file pone.0031878.s002.docx]

**Table S2. Univariate regressions on physical activity counts with initial p < 0.3 to be considered for multiple regression models**

| Variable | | | | R^2^ | Regression Coefficient | | Significance |
| --- | --- | --- | --- | --- | --- | --- | --- |
|  |  |  |  |  | B | SE |  |
| Gender | | | | 0.02 | -21000.0 | 6765.9 | 0.002 |
| Age | | | | 0.17 | -4550.1 | 441.5 | <0.001 |
| SIMD | | | | 0.10 | 15245.0 | 1995.2 | <0.001 |
| Extended theory of planned behaviour (TPB) | Behavioural beliefs | | | 0.11 | 25671.2 | 3133.0 | <0.001 |
|  | PBC (Self efficacy) | | | 0.14 | 13667.4 | 1458.1 | <0.001 |
|  | Normative beliefs | | | 0.13 | 17598.3 | 1959.6 | <0.001 |
|  | Attitudes | | | 0.10 | 17046.0 | 2231.2 | <0.001 |
|  | Subjective norms | | | 0.07 | 18183.0 | 2815.6 | <0.001 |
|  | Need for support | | | 0.02 | -8805.1 | 2749.7 | 0.001 |
|  | Received support | | | 0.04 | 9041.9 | 2054.8 | <0.001 |
|  | Coping planning | | | 0.01 | 3758.8 | 3136.0 | 0.231 |
|  | Action planning | | | 0.05 | 7967.8 | 1489.9 | <0.001 |
|  | Intentions | | | 0.13 | 12716.2 | 1467.1 | <0.001 |
| Urban / Rural | | | | 0.04 | -11117.5 | 2581.9 | <0.001 |
| Geographical | | | % green space in the residential ward | 0.02 | 315.2 | 127.9 | 0.014 |
|  |  |  | Road distance to grocery shop or supermarket (km) | 0.02 | 20739.7 | 7685.9 | 0.007 |
| Local environment (OPAL) | | | Local area surroundings | 0.03 | 3564.6 | 935.6 | <0.001 |
|  |  |  | Streets in your area | 0.01 | 1626.9 | 1378.1 | 0.238 |
|  |  |  | Personal safety | 0.08 | 5281.6 | 768.8 | <0.001 |
|  |  |  | Social contact | 0.01 | 905.4 | 459.9 | 0.049 |
|  |  |  | Overall satisfaction with your local area | 0.01 | 3743.6 | 2021.1 | 0.065 |
|  |  |  | Social support | 0.01 | -2372.5 | 1718.3 | 0.168 |
| Your Health and Wellbeing | | | Physical functioning | 0.25 | 1719.8 | 128.8 | <0.001 |
|  |  |  | Role – Physical | 0.06 | 729.7 | 128.9 | <0.001 |
|  |  |  | Bodily pain | 0.04 | 558.1 | 126.3 | <0.001 |
|  |  |  | General health | 0.09 | 1093.4 | 155.6 | <0.001 |
|  |  |  | Vitality | 0.10 | 1199.3 | 156.5 | <0.001 |
|  |  |  | Social Functioning | 0.04 | 711.5 | 148.3 | <0.001 |
|  |  |  | Role – Emotional | 0.01 | 527.6 | 295.1 | 0.074 |
|  |  |  | Mental Health | 0.02 | 802.4 | 254.1 | 0.002 |
| Loneliness | | R-UCLA | | 0.02 | 5992.7 | 2644.6 | 0.024 |
| Social Capital Module | | | Neighbourliness | 0.03 | 8531.3 | 2435.2 | <0.001 |
|  |  |  | Satisfactory friendship network | 0.01 | 16423.7 | 6993.6 | 0.019 |
|  |  |  | Social support | 0.01 | 9953.9 | 5242.6 | 0.058 |
|  |  |  | Number of people you can turn to who lives nearby | 0.02 | 31811.5 | 11234.8 | 0.005 |
|  |  |  | Number of people during personal crisis | 0.02 | 45513.5 | 14162.4 | 0.001 |
| HADS | | | Depression | 0.09 | -8459.5 | 1200.2 | <0.001 |
| Functional Limitation Profile (FLP) | | | Physical domain | 0.21 | -304.3 | 25.4 | <0.001 |
|  |  |  | Psychosocial domain | 0.07 | -182.7 | 28.4 | <0.001 |
|  |  |  | Communication | 0.03 | -575.0 | 142.3 | <0.001 |
|  |  |  | Work | 0.038 | -164.8 | 37.9 | <0.001 |
|  |  |  | Grand total | 0.173 | -127.0 | 12.0 | <0.001 |
